# Chromatographic Separation of Vitamin E Enantiomers

**DOI:** 10.3390/molecules22020233

**Published:** 2017-02-04

**Authors:** Ju-Yen Fu, Thet-Thet Htar, Leanne De Silva, Doryn Meam-Yee Tan, Lay-Hong Chuah

**Affiliations:** 1 Nutrition Unit, Product Development and Advisory Services Division, Malaysian Palm Oil Board, 6 Persiaran Institusi, Bandar Baru Bangi, 43000 Kajang, Selangor, Malaysia; fujuyen@gmail.com (J.-Y.F.); dtmy1988@gmail.com (D.M.-Y.T.); 2 School of Pharmacy, Monash University Malaysia, Bandar Sunway, 47500 Subang Jaya, Selangor, Malaysia; thet.thet.htar@monash.edu (T.-T.H.); leannedesilva@gmail.com (L.D.S.)

**Keywords:** Vitamin E, tocopherols, tocotrienols, chromatography, enantiomers

## Abstract

Vitamin E is recognized as an essential vitamin since its discovery in 1922. Most vegetable oils contain a mixture of tocopherols and tocotrienols in the vitamin E composition. Structurally, tocopherols and tocotrienols share a similar chromanol ring and a side chain at the C-2 position. Owing to the three chiral centers in tocopherols, they can appear as eight different stereoisomers. Plant sources of tocopherol are naturally occurring in the form of *RRR* while synthetic tocopherols are usually in the form of all-racemic mixture. Similarly, with only one chiral center, natural tocotrienols occur as the *R*-isoform. In this review, we aim to discuss a few chromatographic methods that had been used to separate the stereoisomers of tocopherols and tocotrienols. These methods include high performance liquid chromatography, gas chromatography and combination of both. The review will focus on method development including selection of chiral columns, detection method and choice of elution solvent in the context of separation efficiency, resolution and chiral purity. The applications for separation of enantiomers in vitamin E will also be discussed especially in terms of the distinctive biological potency among the stereoisoforms.

## 1. Introduction

Vitamin E comprises two families of lipid-soluble compounds mainly tocopherols and tocotrienols. Tocopherols are known as part of the antioxidant defense system due to its scavenging ability against peroxyl radicals especially those derived from polyunsaturated fatty acids [1]. Naturally, tocopherols are found as the main vitamin E constituent in soy bean oil, safflower oil and wheat germ while tocotrienols are principally found in palm oil and rice bran oil [2,3]. As α-tocopherol (α-Toc) has been shown to enrich the plasma in comparison to other tocopherols, it is the most highlighted form in vitamin E research [4,5,6]. Being the minor constituent in vitamin E, tocotrienols are gaining rising attention in recent years owing to its potent biological properties including neuroprotection and anticancer activities [7,8].

**Figure 1:**
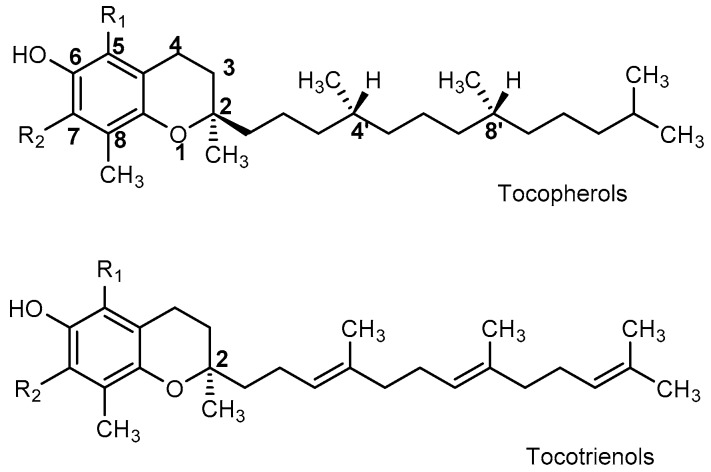
Structures and methyl positions of tocopherols and tocotrienols.

Both tocopherols and tocotrienols share a general core structure composed of, 6-chromanol (benzopyrane) ring system substituted with methyl groups and an isoprenoid side chain. Depending on the variation in the number and positions of methyl substituents on the ring, it can be further classified as α-, β-, γ-, and δ-tocopherols and α-, β-, γ-, and δ-tocotrienols (Figure 1). Briefly, α-forms are trimethylic, the β- and γ-forms are dimethylic and δ-forms are monomethylic. The isoprenoid side chain in tocopherols is represented as a saturated phytyl tail with three stereocenters at C2, C4′ and C8′. Therefore, theoretically, a total of eight optically active stereoisomers can be expected from each α-, β-, γ-, and δ-tocopherols. However, naturally occurring tocopherols are found to have only (2*R*, 4′*R*, 8′*R*) configuration because their biosynthesis that occurs in plants are enantiomerically specific. In contrast, tocotrienols have a single stereocenter with three non-conjugated double bonds in their isoprenoid tails. Naturally occurring tocotrienols are found with (2*R*, 3′*E*, 7′*E*) configuration exclusively. It is worth noting that each component of vitamin E has different strength in antioxidant and biological activities [9].

As the information on health benefits of vitamin E increases, total and semi-synthesis of both α-Toc and α-tocotrienols have been developed to meet the demand of vitamin E. Thus far, the most commonly available form of α-Toc is all-*racemic*-α-Toc (all-*rac*-α-Toc), which is obtained from the reaction of 2,3,5-trimethylhydroquinone with racemic isophytol under acidic condition [10,11,12]. This reaction yields a racemic mixture of α-Toc containing eight stereoisomers in equal proportions. The eight stereoisomers can be grouped into 2*R* configurations (*RRR*, *RSR*, *RRS*, and *RSS*) and 2*S* configurations (*SSS*, *SRS*, *SSR*, and *SRR*). Stereoselective synthesis of α-Toc have produced 2*-ambo*-α-Toc (a mixture of *RRR*- and *SRR*-α-Toc in 1:1 ratio) and 4′*-ambo*-8′*-ambo*-α-Toc (a mixture of 2′-configuration stereoisomers). These products are considered as enantiomerically enriched tocopherol products. Alternatively, using semi-synthetic approach, chemical conversion of natural β-, γ-, δ-tocopherols isolated from a vegetable source to *RRR*-α-Toc (2,5,7,8-tetramethyl-2*R*-(4′*R*,8′*R*,12-trimethyl-tridecyl)-6-chromanol)) via esterification have been attempted to yield *RRR*-α-tocopheryl esters [13].

Due to the chemical stability issues of all-*rac*-α-Toc in free form, more stable ester derivatives such as acetates and succinates have been prepared. Commercially available forms of α-Toc are *RRR*-α-Toc, all-*rac*-α-Toc as well as their esterified derivatives such as all-*rac*-α-tocopheryl acetate (all-*rac*-α-Toc acetate) [1]. With respect to the biopotencies of α-Toc stereoisomers, *RRR*-α-Toc has the highest antioxidant and radical scavenging activities. Higher biopotency of vitamin E activity was observed mainly in 2*R*-enantiomers of α-Toc relative to corresponding *2S*-isomers (i.e., *RRR* = 100%, *SSS* = 60%; *RSS* = 73%, *SRR* = 31%; *RSR* = 57%, *SRS* = 37%; *RRS* = 90%, *SSR* = 21%) [14,15]. The differences in bioactivity and bioavailability of different α-Toc stereoisomers have attracted the interest of many researchers. These studies have also contributed to nutritional recommendations such as the Dietary Reference Intake reported by the Institute of Medicine (IOM) [16]. Based on the distinctive biological activities between the 2*R*- and 2*S*-isomers, the panel has recommended to not include the *2S*-stereoisomeric forms of α-Toc in the dietary allowances for vitamin E. When analyzed in a Irish population, the percentage of plasma *RRR*-isomer was found to be lower in the group taking all-*rac*-vitamin E supplements compared to the group receiving natural vitamin E supplements and the group that was not being supplemented at all [17].

Like tocopherols, total synthesis has led to obtaining racemic products (a total of eight isomers, *RS*, and *E/Z*-) for each tocotrienols. The main challenge in total synthesis of tocotrienols is setting the chirality at position 2. In 1976, the total stereoselective synthesis of *2R*,3′*E*,7′*E*-α-tocotrienol was reported for the first time with the overall yield of 3.44% from trimethylhydroquinone and an average of 84.5% for each of the 20 synthesis steps [18]. After a few decades, Couladouros et al. reported a short and convenient synthesis of optically pure 2-methylchromanmethanols and natural series of β-, γ-, and δ-tocotrienols [19].

In order to understand the distribution of vitamin E stereoisomers in plasma, and tissues, as food additives, various analytical methods have been developed to be able to separate and quantify the stereoisomers. Extensive reviews on chromatographic separation of vitamin E homologues have been done previously [20,21]. However, the main challenge in chromatographic separation of α-Toc stereoisomers remains in the field of chiral resolution. Present review focuses on attempted chromatographic methods, which address the chiral analysis of tocopherol and tocotrienol racemic mixtures obtained from various types of samples.

## 2. Liquid Chromatography

In 1984, Yamaguchi et al. first reported that three different types of α-tocopheryl acetate, namely, natural form of α-Toc (*RRR*-α-Toc), 2-*ambo*-α-Toc and all-*rac*-α-Toc found in different commercial preparations, can be distinguished using high performance liquid chromatography (HPLC) [22]. The authors used commercially available Chiralpak OT (+), a (1)-poly(triphenylmethylmethacrylate)-based as the chiral stationary phase with acetonitrile as the mobile phase set at a flow rate of 0.5 mL/min and ultraviolet–visible (UV) wavelength of 284 nm. The chromatograms obtained showed that *RRR*-α-Toc acetate was eluted as a single peak and 2-ambo-α-Toc acetate was separated into two peaks as it is a mixture of two epimers (Peak 1: *RRR*-α-Toc; and Peak 2: *SRR*-α-Toc). Although all-*rac*-α-Toc acetate is a mixture of eight stereoisomers, the chromatogram obtained showed that it could only be separated into 3 peaks with incomplete racemate separation. The authors concluded that Peak 1 represented a racemate of *RRR* + *SSS* and one more racemate: *RRS* + *SSR* or *RSR* + *SRS*; Peak 2 represented *RRS* + *SSR* or *RSR* + *SRS* and Peak 3 was racemate of *SRR* + *RSS*.

Vecchi et al. saw the weaknesses in this method and hypothesized that the method could be revised in a way that would allow complete separation of (all-*rac*-α-Toc acetate into two peaks containing (2*R*,4′-*ambo*,8′-*ambo*)- and (2*S*,4′-*ambo*,8′-*ambo*)-α-tocopherol, where each peak would contain each of the respective stereoisomers with the same C(2) configuration of the chromanol ring. The authors achieved chromatographic separation of all-*rac*-α-Toc acetate using a self-made stationary steel column with (+) poly(triphenylmethylmethacrylate) ((+)-PTMA) bound to silica gel. Acetonitrile and water (9:1, *v*/*v*) was used as the mobile phase with a flow rate of 0.5 mL/min, and the HPLC system was coupled with UV detection at 200 nm. The chromatogram obtained showed five peaks with their identities determined through the co-injection of authentic stereoisomers, where Peak 1 was *RSR* + *RSS*; Peaks 2 and 3 were *RRR* + *RRS*; Peak 4 was *SSS* + *SSR*; and Peak 5 was *SRS* + *SRR*. This method produced results that were satisfactorily reproducible with a minor drawback that it was time consuming. The authors also reported that the resolution of all-*rac*-α-Toc acetate using the (+)-PTMA column depends greatly on the silica gel pore size, polymerization degree of (+)-PTMA, thickness of the support layer with polymer and temperature. However, the life span of the stationary (+)-PTMA phase was reported to be shorter than chemically-bound stationary phases due to the constant growing of polymers from the mobile phase. It was also noted that after weeks of continuous use of this stationary phase, the selectivity of the column alters itself where the front peaks of all-*rac*-α-Toc acetate gradually moved closer to one another and eventually appears to be no longer separated [23]. This chromatographic method was consequently used by Weiser et al. The authors of the paper also recommended the use of chiral phase Chiracel OD column from Daicel which was more time saving, but it was only available after they had concluded their study.

Ueda et al. had also revised the HPLC method by Yamaguchi et al. for chromatographic separation. This new method allowed for the separation of all-*rac*-α-Toc acetate into four peaks [22,24]. The authors analyzed all-*rac*-α-Toc acetate at 30 °C using a Chiralpak OP (+) column with methanol and water (96:4, *v*/*v*) set at a flow rate of 0.3 mL/min at a UV detection wavelength of 284 nm. The all-*rac*-α-Toc in the biological specimen of rats were found to be divided into four peaks with a peak area ratio of 4:2:1:1 consisting of (*RRR* + *RSR* + *RRS* + *RSS*), (*SSS* + *SSR*), *SRR*, and *SRS*, respectively. The detection limit of stereoisomers in the samples was about 10 ng. The newly revised method by Ueda et al., which allowed complete separation of all-*rac*-α-Toc acetate into 2*R*-isomers and 2*S* isomers, was consequently used by Kiyose et al. in various studies with slight variations in the methanol to H_2_O ratio of the mobile phase [25,26,27].

**Figure 2:**
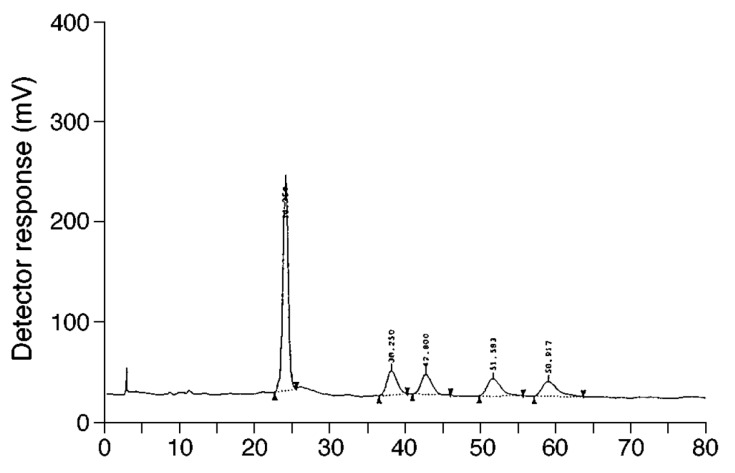
Chiral separation of α-Toc methyl ether by Chiracel OD column. Peak 1: four 2*S*-isomers; Peak 2: *RSS*-; Peak 3: *RRS*; Peak 4: *RRR*; and Peak 5: *RSR* [28]. Reprinted with permission Copyright (1998) by Springer.

Following the development of Chiralcel OD column, a cellulose tris(3,5-dimethyl-phenylcarbamate)-type chiral stationary phase, Nakamura et al. separated the methyl ether derivatives of α-Toc from cellular lipid extracts via chiral phase HPLC Chiralcel OD column. Hexane was used as the mobile phase and was set at a flow rate of 1 mL/min [28]. The UV detector was set at 283 nm to allow detection of the α-Toc methyl ether (α-Toc-ME) isomers. This chiral stationary phase separated all-*rac*-α-Toc-ME derivatives into five peaks (Figure 2) and the whole analysis took 80 min. Peak 1 contained the four *2S*-isomers, which eluted at 25 min; Peak 2 contained *RSS*-isomers (eluted at 38 min); Peak 3 contained *RRS*-isomers (eluted at 43 min); Peak 4 contained *RRR* (eluted at 52 min); and Peak 5 contained *RSR* (eluted at 61 min). The derivatisation of α-Toc to its methyl ether was carried out to block the hydroxyl group, thereby altering the polarity of the analyte and improving its chromatographic properties [29].

While Nakamura et al. performed their analysis using the Chiralcel OD column, Kiyose et al. used the Chiralcel OD-H column. Both the H-series and non-H series columns were reported to have similar selectivity, with the Chiralcel OD-H having superior chromatographic efficiency and overall resolution [15]. Kiyose et al. injected the α-Toc-ME extracted from rat tissue and plasma into an HPLC system equipped with a Chiralcel OD-H using hexane and isopropyl alcohol (97.3:2.7, *v*/*v*) set at a flow rate of 0.3 mL/min and a UV detection wavelength of 268 nm. The first peak on the chromatogram represented *RRR*-α-Toc-ME while the second peak represented *SRR*-α-Toc-ME. Lauridson and Jenson similarly analyzed α-Toc-ME extracted from sow’s milk from lactation and blood using the Chiralcel OD-H column with minor changes in their method, which have been cited by studies analyzing α-Toc from piglet subcutaneous fat and *Longissimus dorsi* muscle [30,31]. Briefly, α-Toc was extracted and derivatised to its methyl ether and injected into an HPLC system equipped with a Chiralcel OD-H using heptane and isopropanol (99.95:0.05, *v*/*v*) as the mobile phase [32]. The fluorescence (FL) detection wavelengths for excitation and emission were set at 295 nm and 330 nm, respectively. The chromatogram showed that the eight stereoisomers were separated into five peaks where Peak 1 contained all four 2*S* forms (2*SR*/*SR*/*S*)-; Peak 2 contained 2*RSS*-; Peak 3 contained 2*RRS*-; Peak 4 contained 2*RRR*-; and Peak 5 contained 2*RSR*-α-Toc. Similarly in a study by Kłaczkow et al. used the Chiralcel OD-H column to separate all-*rac*-α-Toc found in pharmaceutical preparations into their individual stereoisomers [29]. The mobile phase used was hexane set at a flow rate of 1.5 mL/min. The chromatogram showed that α-Toc stereoisomers were separated into five peaks containing eight stereoisomers where Peak 1 contained 2S (*SSR* + *SSS* + *SRS* + *SRR*) stereoisomers (eluted at 12.6 min); Peak 2 contains the *RRS* stereoisomers (eluted at 21.5 min); Peak 3 contains the *RSS* stereoisomer (eluted at 23.3 min); Peak 4 contains the *RRR* stereoisomer (eluted at 27.8 min); and Peak 5 contains the *RSR* stereoisomer (eluted at 32.4 min). Various other studies had been carried out over the years to separate α-Toc into its individual stereoisomers using the Chiralcel OD-H column in chicken feed, liver and thigh [33], cow feedstuffs, muscle, milk and blood [34,35,36] Garcinia Kola seeds [37] and human plasma [17] with minor variations in their mobile phase compositions and FL detections as shown in Table 1.

Chen et al. developed a new chiral stationary phase for open-tubular electrochromatography by immobilizing chitosan nanomaterials onto modified capillaries. The chitosan (CS) nanomaterials were immobilized through copolymerization of glycidyl methacrylate-modified nano-CS with methacrylamide (MAA) and bis-acrylamide crosslinkers therefore resulting in the formation of the MAA-CS capillary. In comparison to packed columns (i.e., Chiralpak-OP and Chiralcel OD/OD-H), open tubular columns lack phase ratios. However, the use of open tubular columns is relatively straightforward, which omits the need for laborious manufacturing of any frits necessary for the column creation. The authors evaluated the chiral separation of α-Toc using a racemic all-*rac*-α-Toc solution from pharmaceutical preparations without any derivatisation in order to evaluate the selective chirality ability of their developed MAA-CS phase. The method used was MAA-CS capillary with borate buffer at varying pH equals of 7.5–9.5 modified with 10% *v*/*v* acetonitrile as the background electrolyte. The voltage applied was 10 kV and α-Toc was added through hydrostatic injection and detected at 200 nm. However, the chromatogram produced from this method had only separated the α-Toc into two peaks which corresponded to 2*R* and 2*S* diastereoisomers of α-Toc. While the changes in pH level and presence of acetonitrile as a modifier helped separate the two peaks, the chromatogram obtained was analogous to that obtained in the previous work using chiral poly acrylate and Chiralpak-OT as HPLC stationary phases for the separation of α-Toc stereoisomers [38].

On the other hand, Drotleff et al. sought developing a reliable HPLC method to analyze synthetic tocotrienol isomers to identify the racemic products of tocotrienol. Prior to derivatisation, α-tocotrienol was divided into its *E*/*Z* isomers through preparative HPLC on permethylated β-cyclodextrin (β-PM). β-PM are cyclic oligosaccharides covalently bounded to silica gel. The cyclodextrin ring of β-PM gives it a cyclical structure which can be illustrated as a truncated cone with hydrophobic interior cavities. The separation behavior of the cyclodextrin ring is influenced by the methylated hydrophilic hydroxyl groups found at the open ends of the truncated cone which use inclusion complexes, hydrogen bonding and dipole interactions as separation mechanisms [39]. Commercially available *RS*-α-tocotrienol was injected into an HPLC equipped with a Nucleodex β-PM column using acetonitrile and water (60:40 *v*/*v*) at a flow rate of 6.5 mL/min and UV detection of 230 nm [40]. The chromatogram displayed four peaks which represented the four side-chain isomers of the commercially available *RS*-α-tocotrienol with an elution order of *RS*,*Z*-*Z*-, second and third *RS*,*Z*-*E*- and *RS*,*E*-*Z*- and *RS,E*-*E*-α-tocotrienol as final isomer to be eluted. After *E/Z* isomers were isolated through the separation of the four fractions, which contained a pair of α-tocotrienol enantiomers each, the separated *E/Z*-α-tocotrienol were methylated [39]. Chromatographic separation of α-tocotrienol methyl ether was then carried out on a Chiralcel OD-H column with 0.05% (*v*/*v*) isopropanol in isohexane as the mobile phase and a flow rate of 1.0 mL/min. The FL detection emission and excitation wavelength was set at 339 and 295 nm, respectively. The derivatisation of tocotrienols to their methyl ethers were carried out to block their highly polar hydroxyl groups therefore preventing unspecific associations with the stationary phase’s carbamate site and allowing for interactions with the cellulose on the stationary phase. The authors also evaluated the impact of varying mobile phases on the separation of *RS,E/Z*-α-tocotrienol methyl ether. They found that isohexane (100%) produced a resolution of six broad peaks which could be achieved within 60 min, whereas isohexane and isopropanol (90:10, *v*/*v*) resulted in elution of all eight isomers without separation.

**Figure 3:**
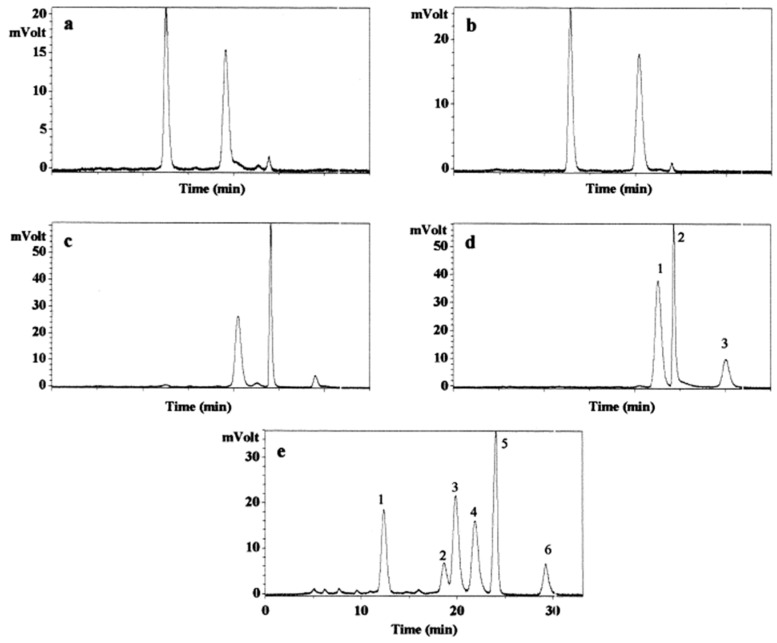
(**a**–**e**) Chromatogram of chiral separation of *E*/*Z*-α-tocotrienol methyl ether by Chiralcel OD-H column into their enantiomers: (**a**) enantiomeric pair of *RS*,*Z*-*Z*-α-tocotrienol methyl ether; (**b**) enantiomeric pair of *RS*,*E*/*Z*-α-tocotrienol methyl ether; and (**c**) enantiomeric pair of *RS*,*E*/*Z*-α-tocotrienol methyl ether. The last peak is presumed to be the degradation product of the secondly eluted enantiomer; (**d**) Peak 1: *S*,*E*-*E*-α-tocotrienol methyl ether; Peak 2: *R*,*E*-*E*-α-tocotrienol methyl ether; and Peak 3 is presumed to represent the degradation product of the Peak 2; (**e**) Chromatogram of chiral separation of *RS,E*/*Z*-α**-tocotrienol methyl ether with omission of preparative HPLC step. Peak 1: diastereomeric pair of *Z*-*Z*- and *E*/*Z*-α-tocotrienol methyl ether; Peak 2: *Z*-*Z*-α-tocotrienol methyl ether enantiomer; Peak 3: diastereomeric pair of *E*/*Z*-α-tocotrienol methyl ether; Peak 4: *S*,*E*-*E*-α-tocotrienol methyl ether; Peak 5: *R*,*E*-*E*-α-tocotrienol methyl ether coeluted with a diastereomer of *E*/*Z*-α-tocotrienol methyl ether; and Peak 6 is presumed to be the degradation product of *R*,*E*-*E*-α-tocotrienol methyl ether [39]. Reprinted with permission. Copyright (2001) by Elsevier.

The chromatograms of *E/Z*-α-tocotrienol methyl ether are shown in Figure 3a–d [39]. The chromatogram showed that the pair of *Z-Z*-α-tocotrienol methyl ether enantiomers had retention times that differed for more than six minutes. The *RS,E/Z*-α-tocotrienol derived from the second peak of the β-PM method showed a distinct separation while the enantiomers of the other *RS,E/Z*-α-tocotrienol derived from the third peak of the β-PM method had retention times which resulted in elution at 3 min intervals. The fourth pair of enantiomers *E-E*-α-tocotrienol methyl ether enantiomers was baseline separated. The authors also found that by excluding the preparative separation of *E/Z* on a β-PM phase before methylation resulted in the eight *RS,E/Z*-α-tocotrienol being separated into five peaks with peak area ratios of 18:5:24:19:27:7 (Figure 3e). Peak 1 on the chromatogram contained two diastereomers of *Z*-*Z*- and *E/Z*-α-tocotrienol methyl ether; Peak 2 contained one enantiomer of *Z*-*Z*-α-tocotrienol methyl ether; Peak 3 contained two diastereomers of *E/Z*-α-tocotrienol methyl ether; Peak 4 contained *S,E,E*-α-tocotrienol methyl ether; Peak 5 contained *R,E,E*-α-tocotrienol methyl ether co-eluted with diastereomer *E/Z*-α-tocotrienol methyl ether; and Peak 6 was assumed to be the degradation product of *R,E,E*-α-tocotrienol methyl ether. To further evaluate the effectiveness of this shortened method, the separation of synthetic β-, γ-, and δ-tocotrienol that also consist of eight possible *RS,E/Z*-isomers were also investigated. *RS*,*E/Z*-β-tocotrienol methyl ether had a long retention time of 42 min. The chromatogram showed nine peaks but due to its low intensity, the fourth peak was assumed to not be an isomer. Whereas, *RS,E/Z*-γ-tocotrienol methyl ether eluted within 10 min and was divided into six peaks and *RS,E/Z*-δ-tocotrienol methyl ether showed separation of all eight isomers.

## 3. Gas Chromatography

**Figure 4:**
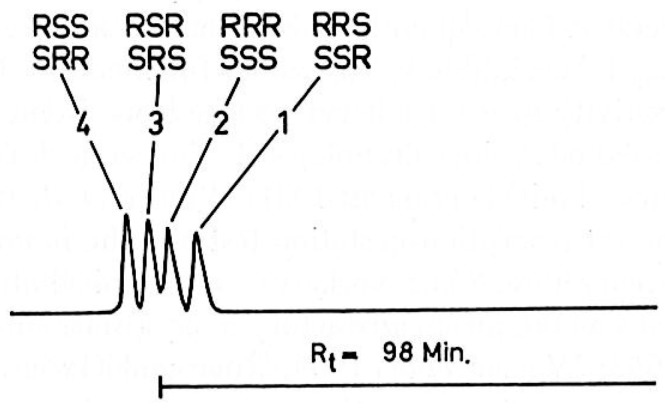
Gas chromatogram of all-*rac*-α-Toc-ME. The four pairs of diastereomers of all-*rac*-α-Toc-ME have equal peak height [44]. Reprinted with permission. Copyright (1981) by Hogrefe.

In 1960s, optical method was used to distinguish *RRR*-α-Toc and all-*rac*-α-Toc [41,42]. The method involved oxidizing the two compounds with potassium ferricyanide and the optical rotations of the products were determined using polarimeter. However, this method only revealed the enantiomeric composition at C2. The first gas chromatographic (GC) technique used to determine the diasteroisomeric composition of natural and synthetic α-Toc samples was developed by Solver and Thompson [42]. In their study, α-Toc samples were derivatised to the corresponding trimethylsilyl (TMS) ether before subjecting to gas chromatography separation. The diastereoisomers of tocopherol TMS ethers were separated on a 115 m × 0.25 mm glass capillary column coated with highly polar liquid phase SP2340, at column temperature of 195 °C. To achieve a better peak resolution, Cohen et al. refined the GC method by converting all-*rac*-α-Toc to the corresponding methyl ethers [43]. The separation was performed on a 100 m × 0.25 mm glass capillary column at 190 °C with Silar 10 C as the stationary phase. This method was adapted by Weiser and Vecchi to analyze different preparations of commercially available α-Toc [14,44]. In addition, Piironen et al. used the same derivatisation method to study the transfer of α-Toc stereoisomers from chicken feeds to eggs [45]. Even though a different column (CP-Sil 88, 50 m × 0.22 mm) was used for the GC separation, the number of peaks detected was the same as that reported by Cohen et al. This is because the column had similar polarity to that of the column used by Cohen et al. All of the reported GC methods utilizing achiral liquid stationary phase and flame ionization detector were able to separate all diastereoisomers of all-*rac*-α-Toc into four distinct peaks with equal in magnitude, in the elution order of peak 1: *RRS* and *SSR*; peak 2: *RRR* and *SSS*; peak 3: *RSR* and *SRS*; peak 4: *RSS* and *SRR* (Figure 4). This indicates that the method can only distinguish the four diastereoisomers but not eight stereoisomers, and the four racemates are present in equivalent amounts. The chromatographic conditions of published GC methods are summarized in Table 2.

## 4. Combined Systems

**Figure 5:**
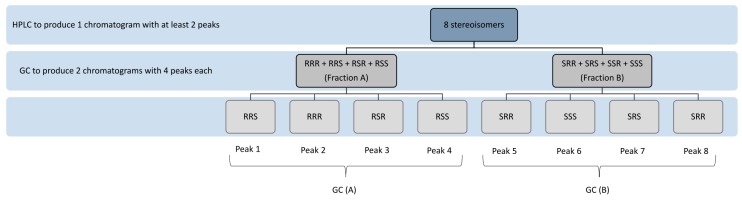
Schematic flow of the separation process for the eight stereoisomers.

A study by Vecchi et al. showed that a combination of chiral HPLC and GC was able to separate all the eight stereoisomers [23]. Instead of using the trimethylsilyl ether [42], methyl ether [43] or acetate form [22] of α-Toc, Vecchi et al. reported the use of ethyl ether form of α-Toc in the separation of the stereoisomers. The schematic flow of the separation process is shown in Figure 5. By using a self-made chiral HPLC stationary phase with (+)-PTMA coated on the silica gel, this method reported a better separation compared to that of Yamaguchi et al. The authors expected four peaks from the HPLC separation, but five peaks were obtained instead, with three peaks having 2*R*-isomers and two peaks having 2*S*-isomers as described earlier in Liquid Chromatography section. For further separation using GC, all the 2R fractions were combined into a single fraction A, and all the 2*S* fractions combined into another fraction B (Figure 5). Fractions A and B were then subjected to GC separation respectively. To calculate the relative intensities of an individual isomer, the relative intensities of the isomer from all four peaks from GC is multiplied by the relative intensities of the corresponding HPLC peak using the following formula (adapted from Vecchi et al.). *SSS* isomer is used as an example here. The same can be applied to calculate the relative proportion of other stereoisomers by substituting the relevant peak area:

(1) $$\frac{F_{\mathrm{GC}\left(B6\right)}}{F_{\mathrm{GC}\left(B5+6+7+8\right)}}\times \frac{F_{\mathrm{LC}\left(B\right)}}{F_{\mathrm{LC}\left(A+B\right)}}\times 100=\%\;\left(S,S,S\right)$$

where F = peak’s surface area; GC (B6) = peak 6 of GC (B) which corresponds to (S,S,S) isomers; LC (B) = peaks B of HPLC; LC (A + B) = sum of all the peaks from both fractions A and B in HPLC; and GC (B5 + 6 + 7 + 8) = sum of all the peaks—5, 6, 7 and 8 in GC (fraction B).

Even though successful separation was obtained for all the stereoisomers, the process of going through two chromatographic events is tedious. The authors hope that eventually an HPLC method (without combination with GC) to separate all eight stereoisomers would be developed [23].

Riss et al. also reported a combination of capillary gas chromatography (GC) method and HPLC system to separate all eight α-Toc stereoisomers in rat tissues and plasma after oral all-*rac*-α-Toc treatment [46]. Before separation begins, the α-Toc was extracted and purified from the tissues and plasma, then converted to α-Toc-ME. The stereoisomers were then subjected to separation with chiral HPLC, followed by capillary GC. Two types of chiral HPLC columns were reported here, namely the self-made Nucleosil 1000-5 column coated with (+)-PTMA (250 × 4 mm) as reported by Vecchi et al., and the commercially available Chiralcel OD column (250 × 4.6 mm) from Daicel. The Chiralcel OD column was a time-saving alternative without the hazard of packing one’s own column. Upon separation by the chiral phase HPLC, 5 peaks were obtained: the first peak consists of all 2S stereoisomers (*SSR* + SSS + *SRS* + *SRR*), while the following four peaks were identified as *RSS*, *RRS*, *RRR* and *RSR*, respectively. Next, 2*S* stereoisomers mixture was subjected to separation using the capillary GC method reported earlier by Vecchi et al. or Cohen et al. [23,43]. The separation was done in the order of SSR, SSS, SRS and SRR. This study showed that the eight stereoisomers could be separated from all-*rac*-α-Toc extracted and purified from tissues and plasma.

In 1996, Weiser et al. investigated the biodiscrimination of all α-Toc stereoisomers in the tissues and plasma of rats dosed with all-*rac*-α-Toc acetate or *RRR*-α-Toc acetate [47]. Similar method of separation as Vecchi et al. was used. Here the Chiralcel OD from Daicel completely replaced the time-consuming self-made column. The samples of the stereoisomers were extracted and purified from the tissues and plasma of rats. α-Toc stereoisomers were recovered. They were then acetylated to α-Toc acetate before being subjected to separation with the chiral HPLC system. At this stage, four peaks were obtained, with peaks 1 and 2 containing 2*R* stereoisomers (*RSR* + *RSS*, *RRR* + *RRS*) and peaks 3 and 4 containing 2S stereoisomers (*SSS* + *SSR*, *SRS* + *SRR*). The samples collected were converted from -α-Toc acetate to -α-Toc-ME, and injected separately into a capillary GC system [23] as all-*R*-*rac* and all-*S*-*rac* mixtures of -α-Toc-ME. All eight stereoisomers were successfully separated into individual stereoisomers. The authors reported successful biodiscrimination of all eight α-Toc stereoisomers in the tissues and plasma of rats after oral supplementation of all-*rac*-α-Toc acetate in vitamin E-depleted rats. This work can be seen as a continuation from those reported by Vecchi et al. and Riss et al., with the 2*R* and 2*S* peaks collected and subjected to further separation with capillary GC. Two other alternative GC systems were also reported here: 100 m × 0.25 mm cyano silicon oil [43] or 50 m × 0.22 mm fused silica capillary column [45]. All combination methods reported in the are summarized in Table 3.

## 5. Applications

The evolution of separation techniques and the ability to quantify stereoisomers of vitamin E, including tocopherols and tocotrienols, had substantially facilitated our understanding of their physicochemical properties and biological discrimination. Back in 1982, the biopotencies of all-*rac*-α-Toc acetate and *RRR*-α-Toc acetate were established at a ratio of 1:1.36 based on conventional rat resorption-gestation tests [14]. This ratio has been widely adopted in the area of animal feed and human nutrition. With the advancement in chromatographic separation methods, more information on their bioavailability has been revealed. As such, results from bioavailability studies can be correlated with their potencies, adding perspective to the subject of interest. For example, in rats supplemented with all-*rac*-α-Toc acetate (100 mg/kg), concentrations of 2R-isomers were markedly higher than the concentrations of the 2S-isomers in plasma, red blood cells, brain, liver, adrenal glands and adipose tissues [24]. Similarly, when all-*rac*-α-Toc acetate was supplemented in milk replacer for calves, *RRR*-α-Toc was found to be the dominant stereoisomer in plasma and tissues [48]. While other 2*R*-isomers have lower absorption rates than the RRR-isomer, the *2S*-isomers were basically not utilized by calves. The results were in agreement with IOM recommendation that 2*S*-isomers are likely to be under-utilized in the biological systems. In a separate study where rats were supplemented with increasing doses of all-*rac*-α-Toc acetate or *RRR*-α-Toc acetate, mean apparent absorption coefficient from *RRR*-source was higher than all-*rac*-source [49]. An increasing trend from 77.2 to 83.3 in the absorption coefficient of all-*rac*-source was observed with increasing doses from 25 to 200 mg/kg feed while RRR-source reached an average of 86.8 absorption coefficient at a dose as low as 25 mg/kg feed. In fact, natural α-Toc in micellized form supplemented at 1/3 the dose of synthetic α-Toc resulted in similar plasma α-Toc concentrations in piglet study [30]. When investigated in humans, supplementation of *RRR*-α-Toc at 100 mg/day resulted in similar serum α-Toc levels compared to 300 mg/day all-*rac*-α-Toc acetate, correlating to 1/3 of a dose difference [27]. The study also showed that only small amount of 2*S*-isomers were detected in the serum, which were significantly lower than the 2*R*-isomers.

From a mechanistic point of view, high density lipoprotein (HDL) was found to exhibit the highest donor capacity for α-Toc when investigated in rat skeletal muscle cells in the presence of lipoproteins [28]. Cells incubated at equipotent doses of all-*rac*- and *RRR*-α-Toc (1.36:1) did not show significant biodiscrimination in the uptake of 2*S*- and 2*R*-isomers, i.e., 2*S*- and 2*R*-isomers were accumulated at 1:1 ratio. From this study, the authors postulated that the in vivo biodiscrimination of α-Toc was attributed to plasma enrichment with *RRR*-isomer instead of mechanisms at the cellular level. Studies involving the use deuterium-labeled *RRR*- and *SRR*-α-Toc acetate revealed a similar trend where the discrimination between the stereochemistry did not occur during absorption but rather as a post-absorptive phenomenon in the liver [50]. This was supported by the fact that chylomicrons contained similar concentrations of the *RRR*- and *SRR*-isomers, while plasma and very low density lipoproteins (VLDL) strongly discriminates the natural vs. synthetic stereochemistry.

In addition to biodiscrimination and mechanistic studies, separation of vitamin E stereoisomers had also been used as a reliable method to distinguish the different sources of pigs from controlled farms [31]. By analyzing the pig fat samples, higher presence of *RRR*-isomer contributed by natural form of vitamin E indicated pig feed from natural sources, highest in the FREE-RANGE category, followed by FREE-FEED, FEED-OUT and FEED. Results from the study also helped to identify if supplementation in the feeds were contributed by natural oil ingredient or synthetic form of vitamin E. In another animal feed study, quantification of α-Toc stereoisomer distribution in porcine liver tissues helped to identify the interaction between dietary fatty acid composition and vitamin E absorption [51]. The study postulated that the demand for *RRR*-isomer of α-Toc as anti-oxidant is directly correlated with the degree of unsaturation in dietary fat. 

## 6. Conclusions

Differences in bioavailability and biopotencies of individual stereoisomers of tocopherols and tocotrienols have led to the importance of chiral separations for pharmaceutical related studies. Challenge in chiral resolution is generally attributed by enantiomers character of having identical physical and chemical properties. Having a single stereocenter in tocotrienols, a complete separation of a racemic mixture of β- and δ-tocotrienols has been successfully achieved using chiral HPLC (3.5-dimethyl phenyl carbamate) method in a single analytical system [39]. However, the separation of the eight stereoisomers of α-Toc, so far, requires a combination of at least two different analytical methods, mainly HPLC and GC. The HPLC analysis of both tocopherols and tocotrienols requires derivatisation to corresponding esters or ethers to prevent degradation of the compounds before eluting.

Due to the limitations in the analytical system to provide complete separation of α-Toc stereoisomers, the distribution of all eight stereoisomers in tissues cannot be measured [47]. Lately, the combination of GC and mass spectrometry (MS) has allowed quantification of deuterium labeled tocopherols in blood and tissues [52,53]. One of the advantages of this method was that absorbed labeled vitamin E molecules can be distinguished from the endogenous vitamin E. In this way, the investigator can quantify the distribution of labeled α-Toc in various tissues. However, this technique does not provide the information on the quantity of the eight stereoisomers.

Recently, technologies in chromatographic columns and analytical systems have largely advanced. Further studies in separation of the eight α-Toc stereoisomers from various sample matrices in a single analytical system (chiral HPLC) can be explored using electrochemical detection which is more sensitive than commonly used UV absorbance or FL detection. Literature survey done by Kumar et al. revealed that MS technology has been significantly improved by advancing various ionization techniques including electrospray ionization (ESI), and atmospheric pressure ionization (API) [54]. During the last few years, several chiral analysis studies of pharmaceuticals have been performed using enantiomeric liquid chromatography coupled to tandem mass spectrometry (LC-MS) [55,56,57]. The LC-MS technique is based on a combination of the resolving power of HPLC using improved chiral stationary phases and superior mass spectrometric detection systems operated by API source. This application has been proven to be more reliable in comparison to GC-MS method in the determination of enantiomeric composition of methamphetamine in urine sample [58].

In addition to LC-MS approach, there exist other approaches of mass-selective chiral analysis using MS. One of them is the analysis performed by using the ion source such as resonance-enhanced multiphoton ionization (REMPI), which is linked to circularly polarized light, electronic circular dichroism (ECD) in a mass spectrometer. It is based on UV spectrum of the ionized molecule, resulting in improved mass selectivity, sensitivity and speed. Recently, photoelectron circular dichroism (PECD) technique has been developed to differentiate enantiomers via particle imaging. When a chiral molecule is photoionized by a circularly polarized femtosecond laser, the spatial angular distribution of photoelectrons emitted by ionization of the chiral analyte can be obtained using imaging techniques [59]. This technique has been further advanced to combine with photoion coincidence (PEPICO) spectroscopy to obtain mass-selected PECD [60]. Boesl and Kartouzian have reviewed extensively on mass-selective chiral analysis and have noted that PECD combined with PEPICO is a promising method to study molecular chirality [61]. Therefore, chiral analysis of tocopherols and tocotrienols can be further explored with the application of advanced MS technologies.

## Figures and Tables

**Table:** 

Tocopherols	Tocotrienols	R_1_	R_2_
α-Tocopherol	α-Tocotrienol	CH_3_	CH_3_
β-Tocopherol	β-Tocotrienol	CH_3_	H
γ-Tocotrienol	γ-Tocotrienol	H	CH_3_
δ-Tocopherol	δ-Tocotrienol	H	H

**Table 1:** HPLC methods for α-Toc and α-, β-, γ-, and δ-tocotrienol separation.

Column	Mobile Phase	Analytes	Detection Wavelength	Stereoisomer/Diastereomers Separation	Application	Reference
Chiralcel OD-H (250 × 4.6 mm I.D.)	*n*-hexane	α-Toc methyl ether	FL: 284 nm (Ex)	(*SSS* + *SSR* + *SRR* + *SRS*), *RSS*, *RRS*, *RRR*, *RSR*	Cow feed and muscle, human plasma	[17,36]
			326 nm (Em)			
Chiralpak OP (+) (250 × 4.6 mm I.D.)	Acetonitrile	α-Toc acetate	UV: 284 nm	(*RRR* + *SSS*, *RRS* + *SSR*, *RSR* + *SRS*), (*RRS* + SSR, *RSR* + *SRS*), (*SSR* + *RSS*)	Commercial product	[22]
Nucleosil1000-5 coated with (+)-PTMA) (250 × 4 mm I.D.)	Acetonitrile/H_2_O	α-Toc acetate	UV: 200 nm	(*RSR* + *RSS*), (*RRR* + *RRS*), (*SSS* + *SSR*), (*SRS* + *SRR*)	Commercial product, rat blood and tissue	[23]
Chiralpak OP (+) (250 × 4.6 mm I.D.)	Methanol/H_2_O	α-Toc acetate	UV: 284 nm	(*RRR* + *RSR* + *RRS* + *RSS*), (*SSS* + *SSR*), *SRR*, *SRS*	Rat tissue, blood, plasma and tissue, human serum and lipoproteins	[24,25,26,27]
Chiralcel OD (250 × 4.6 mm I.D.)	*n*-hexane	α-Toc methyl ether	UV: 283 nm	(*SSS* + *SSR* + *SRR* + *SRS*), *RSS*, *RRS*, *RRR*, *RSR*	Cellular lipid extracts	[28]
Chiralcel OD-H (250 × 4.6 mm I.D.)	*n*-hexane	α-Toc methyl ether	FL: 295 nm (Ex)	(*SSS* + *SSR* + *SRR* + *SRS*), *RSS*, *RRS*, *RRR*, *RSR*	Pharmaceutical preparations of Vitamin E	[29]
			330 nm (Em)			
Chiralcel OD-H (250 × 4.6 mm I.D.)	*n*-heptane/isopropanol	α-Toc methyl ether	FL: 290 nm (Ex)	(SSS + *SSR* + *SRR* + *SRS*), *RSS*, *RRS*, *RRR*, *RSR*	Pig milk from lactation, blood, subcutaneous fat and piglet Longissimus dorsi muscle, rat plasma, tissues and faeces, cow milk and blood	[30,31,32,34]
			327 nm (Em)			
Chiralcel OD-H (250 × 4.6 mm I. D.)	*n*-heptane /isopropanol	α-Toc methyl ether	FL: 295 nm (Ex)	(*SSS* + *SSR* + *SRR* + *SRS*), *RSS*, *RRS*, *RRR*, *RSR*	Chicken feed, liver and thigh	[33]
			330 nm (Em)			
Chiralcel OD-H (250 × 4.6 mm I.D.)	*n*-heptane/isopropanol	α-Toc methyl ether	FL: 296 nm (Ex)	(*SSS* + *SSR* + *SRR* + *SRS*), *RSS*, *RRS*, *RRR*, *RSR*	Cow plasma, colostrum, milk and blood neutrophils	[35]
			372 nm (Em)			
Chiralcel OD-H (250 × 4.6 mm I.D.)	Hexane/ethanol	α-Toc	UV: 220 nm	(*SSS* + *SSR* + *SRR* + *SRS*), (*RSS* + *RRS* + *RRR* + *RSR*)	Garcinia Kola seeds	[37]
MAA-CS capillary (52 cm (47 cm) × 75 mm I.D.)	Background electrolyte: borate buffer modified with acetonitrile	α-Toc	UV: 220 nm	(*SSS* + *SSR* + *SRR* + *SRS*), (*RSS* + *RRS* + *RRR* + *RSR*)	Pharmaceutical preparation of Vitamin E	[38]
Chiralcel OD-H (250 × 4.6 mm I.D.)	Isohexane/isopropanol	α-tocotrienol	FL: 295 nm (Ex)	(*RS*,*Z-Z*), (*RS*,*Z-E*-), (*RS*,*E-Z*-), (*RS*,*E*-*E*)	Pharmaceutical preparation of Vitamin E	[39]
			339 nm (Em)			
Nucleodex β-PM (200 × 4 mm I.D.)	Acetonitrile/H_2_O	α-tocotrienol	UV: 230 nm	(*RS*, *Z*-*Z*- + *RS*,*E*/*Z*- diastereomer), (*RS*,*Z*-*Z*- enantiomer), (*RS*,*E*/*Z*-diasteromers ), (*RSS*,*E-E*), (*RSR*,*E-E* + *RS*,*E*/*Z*- diastereomer)	Pharmaceutical preparation of Vitamin E	[40]

UV, Ultraviolet detection; FL, Fluorescence detection; Ex, excitation; Em, Emissio.

**Table 2:** GC methods for α-Toc separation.

Column	Carrier Gas	Injector Condition	FID Temperature	Analytes	Stereoisomer/Diastereomers Separation	Application	Reference
100 m × 0.3 mm glass capillary column coated with *Silar* 10 C; temperature 185 °C	Hydrogen at 25 cm/s; split ratio 1/50	Sample concentration 1 mg/mL; temperature 270 °C	270 °C	Different preparations of all-*rac*-α-tocopheryl acetate from large scale production, 4’-*ambo*-8’-*ambo*-α-tocopheryl acetate, RRR-α-tocopheryl acetate. All samples were derivatised into methyl ethers.	(*RRS* + *SSR*), (*RRR* + *SSS*), (*RSR* + *SRS*), (*RSS* + *SRR*)	Commercial product	[14]
Glass capillary column (115 m × 0.25 mm, coated with highly polar liquid phase SP2340; temperature 195 °C)	Hydrogen at 19 cm/s; split ratio 1/50 to 1/100	Sample size 1.7 μL at concentration 2 mg/mL; temperature 280 °C	300 °C	*RRR*-α-tocopherol, 2-*ambo*-α-tocopherol, all-*rac*-α-tocopherol and 4’-*ambo*-8’-*ambo*-α-tocopherol. All samples were derivatised into TMS ethers.	2-*ambo*-α-tocopherol: *RRR*, *SRR*. All-*rac*-α-tocopherol: (*RRR* + *SSS*), (*RSS* + *SRR*). 4’-*ambo*-8’-*ambo*-α-tocopherol: *RRR*, *RSS*	Commercial products	[42]
Glass capillary column (100 m × 0.3 mm, coated with *Silar* 10 C; temperature 185 °C)	Hydrogen at 17cm/s; split ratio 1/200	Sample size 2 μL at concentration 1 mg/mL; temperature 250 °C	300 °C	All-*rac*-α-tocopherol and α-tocopheryl acetate. All samples were derivatised into methyl ethers.	(*RRS* + *SSR*), (*RRR* + *SSS*), (*RSR* + *SRS*), (*RSS* + *SRR*)	Commercial products	[43]
100 m × 0.3 mm glass capillary column coated with *Silar* 10 C; temperature 185 °C	Hydrogen at 25 cm/s; split ratio 1/50	Sample concentration 1 mg/mL; temperature 270 °C	270 °C	All-*rac*-α-tocopheryl acetate, 2-*ambo*-α-tocopheryl acetate, RRR-α-tocopheryl acetate. All samples were derivatised into methyl ethers.	(*RRS* + *SSR*), (*RRR* + *SSS*), (*RSR* + *SRS*), (*RSS* + *SRR*)	Commercial product	[44]
Fused silica capillary column (50 m × 0.22 mm, CP-Sil 88; temperature programmed from 150 to 210 °C at 2 °C/min) (with a 2-min hold at 150 °C and 10 min hold at 210 °C) and from 210 to 230 °C at 1 °C/min (with 20 min hold at 230 °C)	Helium at 1.8 mL/min; split ratio 1/30	Sample size 0.7–2.0 μL; temperature 240 °C	260 °C	α-tocopherol in chicken feed and eggs, derivatised into methyl ethers.	(*RRS* + *SSR*), (*RRR* + *SSS*), (*RSR* + *SRS*), (RSS + *SRR*)	Animal feeds and products	[45]

FID, flame ionization detector.

**Table 3:** Combination methods (HPLC-GC) for α-Toc separation.

Analytes	HPLC			GC			Stereoisomer/Diastereomers Separation	Application	Reference
	Column	Mobile Phase	Detection Wavelength	Column	Injector Condition	FID Temperature			
α-Toc ethyl ether	Nucleosil1000-5 coated with (+)-PTMA) (25 × 0.4 cm)	Acetonitrile/H_2_O (9:1, *v*/*v*)	200 nm (UV)	Silar 10 C coated glass capillary tube (100 m × 0.30 mm), isothermal at 165 °C	Splitless mode at 260 °C	220 °C	*RRS, RRR, RSR, RSS, SRR, SSS, SRS, SRR*	Commercial product	[23]
α-Toc acetate (HPLC), α-Toc methyl ether (GC)	Chiralcel OD (25 × 0.46 cm)	Acetonitrile/H_2_O (9:1, *v*/*v*)	200 nm (UV)	Silar 10 C coated glass capillary tube (100 m × 0.30 mm), isothermal at 165 °C	Splitless mode at 260 °C	220 °C	*RRS, RRR, RSR, RSS, SRR, SSS, SRS, SRR*	Rat blood and tissue	[47]
α-Toc methyl ether	Nucleosil1000-5 coated with (+)-PTMA) (25 × 0.4 cm) Chiralcel OD (25 × 0.46 cm)	*n*-hexane	200 nm (UV)	Silar 10 C coated glass capillary tube (100 m × 0.30 mm), isothermal at 165 °C	Splitless mode at 260 °C	220 °C	*RRS, RRR, RSR, RSS, SRR, SSS, SRS, SRR*	Rat blood and tissue	[46]

FID, flame ionization detector.
